# Real‐world experience of rIX‐FP prophylaxis at dosing intervals of up to 14 days in a pediatric patient with hemophilia B during the COVID‐19 pandemic

**DOI:** 10.1002/ccr3.8180

**Published:** 2023-11-28

**Authors:** Bolívar Luis Díaz‐Jordán, Tamara Cebanu, Sara García Barcenilla, Maria Teresa Álvarez‐Román

**Affiliations:** ^1^ Hematology Department Hospital General de Valdepeñas Ciudad Real Spain; ^2^ Hematology and Hemostasis Department Hospital Universitario La Paz Madrid Spain; ^3^ Hospital La Paz Institute for Health Research – IdiPAZ (Hospital Universitario La Paz – Universidad Autónoma de Madrid) Madrid Spain

**Keywords:** hemophilia B, pediatrics, prophylaxis, rIX‐FP

## Abstract

Switching to rIX‐FP prophylaxis at dosing intervals of up to 14 days in a hemophilia B pediatric patient decreased treatment burden by reducing the number of administrations and hospital visits, without affecting efficacy or treatment adherence. This is particularly important in contexts of limited mobility and overloaded healthcare services.

## 1 INTRODUCTION

Prophylactic regimens have become the standard of care for hemophilia B patients, although patient adherence to these therapies often represents a barrier, particularly in pediatric patients, due to the need for frequent intravenous injections. Recombinant factor IX albumin fusion protein (rIX‐FP, albutrepenonacog alfa, IDELVION®), an extended half‐life recombinant factor IX (EHL FIX), has shown good prophylactic efficacy in clinical trials of treatments administered up to every 21 days in adult patients, and up to every 14 days in selected pediatric patients with hemophilia B.^1^ The prolonged half‐life of rIX‐FP and pharmacokinetic properties are achieved by the fusion with recombinant albumin, a natural, inert carrier protein in plasma with a half‐life of approximately 20 days. Unlike other products, rIX‐FP remains intact in the bloodstream until FIX is activated, and thereupon albumin is cleaved, releasing activated FIX when it is needed for coagulation.^2^ Currently, rIX‐FP is approved for once‐weekly prophylaxis in pediatric patients with hemophilia B.

Here we report a real‐world case of a pediatric hemophilia B patient who switched from standard rFIX (nonacog alfa), a standard half‐life FIX (SHL FIX), to rIX‐FP prophylaxis during the COVID‐19 pandemic.

## 2 CASE PRESENTATION

A 4‐year‐old patient previously diagnosed with moderate hemophilia B (FIX = 2.7 IU/dL) presented in May 2018 with spontaneous hemarthrosis in the left knee. The patient began prophylactic treatment with rFIX (40 IU/kg) twice weekly, as he met the criteria for prophylaxis instead of on‐demand treatment according to the international guideline's recommendations.^3^ This regimen lasted for almost 2 years, although due to difficulties with venous access, factor injections had to be administered in our center, which is 45 km away from the family home. Clinical evaluations were regularly performed to guide patient management. Hemophilia Early Arthropathy Detection with Ultrasound (HEAD‐US) score was 0, a genetic study revealed a nonsense mutation on the *F9* gene (1436A < T), FIX level was 2.7%, and the patient had no family history of bleeding diathesis.

A rFIX‐FP pharmacokinetic profile performed using WAPPS‐Hemo V.2.03 showed an extended half‐life for rFIX‐FP (mean half‐life of 125.5 h with 50 IU/kg of product) and a prolonged activity time (15 days) to reach FIX levels of 5%. Given these results and the available published evidence,^1^ the patient was switched to weekly rIX‐FP prophylaxis (40 IU/kg) in March 2020, in view of mobility difficulties associated with the beginning of the COVID‐19 pandemic. Treatment was well tolerated after the first dose with rIX‐FP, and pharmacokinetics (PK) testing revealed postinfusion trough levels at 14 days and 20 days of 7.7 IU/dL and 5.1 IU/dL, respectively. Functional examination and joint assessment on day 90 of rIX‐FP treatment did not show any significant change compared to baseline, no FIX inhibitors had developed, while the treatment burden was lower due to the reduced number of administrations and fewer hospital visits. On day 95 of treatment, the patient presented a post‐traumatic otorrhagia of the left ear (due to laceration of the external auditory canal by a cotton bud), which resolved after a single 40 IU/kg dose of rIX‐FP.

**FIGURE 1:**
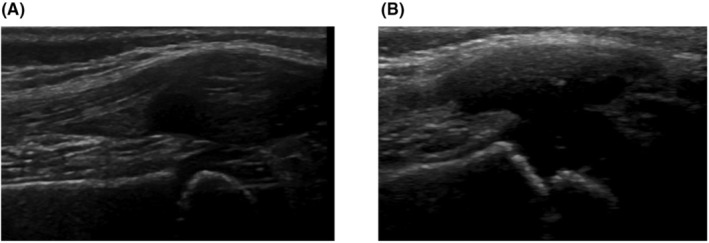
Images of the HEAD‐US evaluation of the right knee performed in 2020 (A) and 2021 (B). There are no signs of joint effusion in the suprapatellar recess. Articular cartilage with no changes in morphology and echogenicity and periosteum with no changes in echogenicity.

The clinical evaluation on day 120 of treatment showed no change in joint status. For this reason, the administration regimen was extended to 40 IU/kg every 10 days. On day 160 of treatment, the patient was diagnosed with asymptomatic COVID‐19 and self‐isolated at home for 14 days following the recommendations of the health authorities at that moment. During this period, treatment with rIX‐FP remained unchanged. After 200 days of treatment, a PK study confirmed trough levels of 6.9 IU/dL at 14 days postinfusion. Prophylactic treatment was subsequently extended to 70 IU/kg every 14 days, and the patient has presented no bleeds or complications to date. Joint evaluations, performed annually, gave a Hemophilia Joint Health Score (HJHS) and HEAD‐US score of 0, and no signs of joint disease were detected (Figure 1).

After switching to a 14‐day rIX‐FP regimen, monthly consumption fell by 56.2% and the number of infusions by 75%, compared to the initial rFIX treatment. This switch, moreover, did not affect the patient's physical activity and had no impact on treatment efficacy and adherence.

## 3 DISCUSSION

There is ample evidence that extended half‐life (EHL) products have half‐lives that are 3–6 times longer than standard half‐life (SHL) products and offer the possibility of fewer injections. rIX‐FP is approved for starting once‐weekly prophylaxis in adult and pediatric patients with hemophilia B. For patients ≥12 years, further extension of the treatment interval up to 14 days may also be considered, and up to 21 days for patients >18 years.^2^

Our case demonstrates that switching from prophylaxis with SHL FIX to EHL rIX‐FP at dosing intervals of up to 14 days is an optimal approach in pediatric hemophilia B patients. This is in line with recent real‐world data showing the benefits of this switch in adults and children with hemophilia B from different countries.^4^ Benefits include a reduction in annual bleeding rates (ABR), more patients with zero total and spontaneous bleeds, and a dosing interval of longer than a week (reducing weekly consumption). Steady trough factor levels were maintained in our patient during the treatment course, as previously reported.^1^ The successive extensions of the prophylactic regimen did not interfere with our patient's treatment adherence. In fact, higher adherence rates may be achieved with extended dosing intervals,^5^ resulting in better clinical outcomes, prognosis, and patient quality of life (QoL).

We are aware that the SETH (*Spanish Society of Thrombosis and Hemostasis*) recommended against switching FIX products during the COVID‐19 pandemic, but this patient switched before the publication of these guidelines. Nevertheless, in view of our patient's outcome, the recommendations could perhaps be individualized depending on the clinical, geographic, pharmacokinetic, and/or sociosanitary context.

Our patient's young age prevented the implementation of a validated QoL questionnaire. However, we clearly perceive a positive impact if we take into account the reduction of hospital visits, the absence of hospital admissions, the clinical and PK data collected (ABR = 0, HEAD‐US = 0, HJHS = 0), and savings in school/working days (for the patient and the parents).

The outcomes from this clinical case are in line with those recently observed in a similar pediatric hemophilia B patient from Spain.^6^ Furthermore, our case highlights how this management pathway should be especially considered for any patient with difficult access to the health center or in the context of the COVID‐19 pandemic. A reduced treatment burden in terms of fewer trips, hospital visits, and injections is important, not only from the perspective of the patient and their family, but also for the hospitals in terms of saved time in the hematology department for other tasks.

## AUTHOR CONTRIBUTIONS

**Bolívar Luis Díaz‐Jordán:** Conceptualization; formal analysis; investigation; writing – review and editing. **Tamara Cebanu:** Formal analysis; investigation; writing – review and editing. **Sara García Barcenilla:** Formal analysis; investigation; writing – review and editing. **Maria Teresa Álvarez‐Román:** Conceptualization; formal analysis; investigation; writing – review and editing.

## FUNDING INFORMATION

This service was funded by CSL Behring.

## CONFLICT OF INTEREST STATEMENT

BLDJ has received honoraria for speaking engagements or funds for research from CSL Behring, Takeda, NovoNordisk, Roche, Daiichi‐Sankyo, Sobi, Fresenius, Leo Pharma, Abbvie, and Janssen. TC has no conflicts of interest to declare. SGB has received honoraria for speaking engagements from Roche, NovoNordisk, CSL Behring, Takeda, Sobi, Biomarin, Novartis, Bayer and Pfizer. MTAR has received honoraria for speaking engagements or consultancy, or funds for research from Takeda, Bayer, CSL Behring, Grifols, Novo Nordisk, Sobi, Octapharma, Roche, Amgen, Novartis, and Pfizer.

## CONSENT

Written informed consent was obtained from the patient's parents to publish this report in accordance with the journal's patient consent policy.

## Data Availability

Data available on request from the authors.
